# Enhanced stability and reusability of metagenomic laccase via immobilization on functionalized mesoporous silica for antibiotic contaminant removal

**DOI:** 10.1038/s41598-026-40065-w

**Published:** 2026-03-26

**Authors:** Shohreh Ariaeenejad, Sedigheh Abedanzadeh

**Affiliations:** 1 Department of Systems and Synthetic Biology, Agricultural Biotechnology Research Institute of Iran (ABRII), Agricultural Research Education and Extension Organization (AREEO), Karaj, Iran; 2 Faculty of Chemistry, Kharazmi University, Tehran, 15719-14911 Iran

**Keywords:** Porous material, Nanocarrier, Metagenomic laccase, Antibiotic removal, Enzyme immobilization, Biotechnology, Environmental sciences, Microbiology

## Abstract

The extensive application of tetracycline antibiotics in agriculture and medicine has led to persistent contamination of aquatic and terrestrial ecosystems, disrupting microbial communities and contributing to the spread of antibiotic resistance. Conventional treatment methods often suffer from poor efficiency, limited stability, and high environmental costs, underscoring the need for robust and sustainable alternatives. Here, we present a biocatalytic platform in which a metagenome-derived laccase (PersiLac1) is covalently immobilized onto imidazole-functionalized SBA-15 mesoporous silica to overcome the limitations of free laccase, including low stability and high leaching. Immobilization markedly enhanced thermal stability, reusability, and catalytic efficiency toward the degradation of doxycycline (DC) and tetracycline (TC). The optimized system exhibited minimal enzyme leaching (9.6% at 25 °C; 22.0% at 80 °C) and achieved removal efficiencies of 76.7 ± 2.8% for DC and 53.7 ± 2.1% for TC within 24 h. High removal performance was maintained even at elevated antibiotic concentrations (200 mg L⁻¹), with 43.9% and 42.8% removal for DC and TC, respectively. The immobilized laccase retained over 83% (DC) and 73% (TC) of its initial activity after 10 consecutive reuse cycles. To the best of our knowledge, this is the first report of integrating a metagenomic laccase with an imidazole-functionalized SBA-15 support for antibiotic degradation, offering a unique combination of enhanced stability, high reusability, and environmentally relevant performance. These findings highlight the potential of this immobilization strategy as a sustainable and high-performance solution for the remediation of antibiotic contaminants in water systems.

## Introduction

Laccases, members of the multi-copper oxidase family, have a wide range of applications in environmental biotechnology, particularly in water bioremediation. These oxidative enzymes catalyze the oxidation of a variety of phenolic and non-phenolic aromatic compounds^1^and play an important role in the digestion and management of lignocellulosic biomass^2^. By removing phenolic inhibitors, laccases improve the accessibility of cellulose to hydrolytic enzymes, thereby enhancing biomass processing efficiency^3–5^. Their versatility has led to applications in decolorization, delignification, detoxification, and pollutant degradation. Tetracycline antibiotics (TCs)- including tetracycline (TC), chlortetracycline (CTC), doxycycline (DC), and oxytetracycline (OTC)- are widely used in human and veterinary medicine. However, their persistence in the environment and potential to promote antibiotic resistance have raised serious concerns^6,7^. A significant fraction of these compounds is excreted unmetabolized and enters wastewater and agricultural runoff, contaminating natural ecosystems^8,9^. This has intensified the need for eco-friendly and effective strategies to remove such pollutants.

Laccases offer a promising enzymatic approach for degrading tetracyclines; however, free laccases often suffer from low stability and poor reusability. Despite various enzymatic studies, few have explored the use of immobilized metagenomic laccases for degrading tetracycline-class antibiotics under environmentally relevant conditions. Moreover, the use of imidazole-functionalized nanomaterials to enhance catalytic performance remains underexplored, representing a significant research gap. These agents are extensively applied to treat bacterial infections and are also used in livestock production, particularly in poultry farming, both for disease prevention and as growth promoters^10^. However, excessive or improper use leads to their release into aquatic and terrestrial environments, as a significant proportion (up to 70% of the administered dose) is excreted unmetabolized^8^. For example, the concentration of DC in poultry manure can reach 33.1 mg/kg (dry weight)^9^. Such residues pose ecological risks, including the promotion of antibiotic resistance, highlighting the urgent need for effective removal strategies. Laccases offer a promising, eco-friendly approach to address this challenge, as they can oxidatively degrade a broad range of organic pollutants, including the structurally complex tetracycline antibiotics^11^. By transforming antibiotics into less toxic or non-toxic products, laccases help mitigate their environmental impact^11^. Nevertheless, free laccases suffer from low operational stability, slow reaction rates, and challenges in recovery and reuse^12^. Immobilization is an effective strategy to overcome these drawbacks, enhancing enzyme stability against pH and temperature variations, improving reusability, and facilitating separation from treated water^13–15^. Laccases can be sourced through metagenomic approaches, which enable direct access to the genetic potential of entire microbial communities without the need for cultivation^16,17^. By combining bioinformatics screening^18^with functional assays, novel and stable laccases adapted to extreme environments can be rapidly identified^14,16,19^.

Ordered mesoporous materials (OMMs) have received much attention as viable supports for enzyme immobilization^20–24^. Among these, SBA-15 is one of the most widely studied materials, characterized by mesopore-sized channels, high surface area, and large pore volume^25–27^. Surface modification of SBA-15 allows precise control over its physicochemical properties, including hydrophilicity/hydrophobicity, which can enhance enzyme-support interactions. Such modifications can lead to higher enzyme loading, improved protection against chemical denaturation, and increased overall stability and activity^28^. Targeted surface modification of SBA-15 would be easily conducted through the reaction of rich silanol groups (≡ Si–OH) located in pore walls of SBA-15 in contact with modifying organic groups^29,30^. For enzyme immobilization, physical adsorption-primarily via hydrogen bonding, van der Waals forces, hydrophobic effects, and electrostatic interactions- offers a simple and gentle loading method. However, this approach often suffers from weak interactions, leading to significant enzyme leaching and reduced long-term stability. In contrast, chemical immobilization involves covalent bonding between the enzyme and the support, producing highly stable biocatalysts with superior resistance to leaching.

In recent years, our main research interest is focused on designing eco-friendly waste management strategies to achieve greener environment^31,32^. Recognizing the pivotal role of laccases in degrading antibiotic contaminants, the present study investigates the laccase-mediated degradation of tetracycline antibiotics.

In this study, we utilized an imidazole-functionalized SBA-15 as a carrier for immobilization of a metagenome-derived laccase (PersiLac1)^19^, aiming to enhance its catalytic performance for TC and DC degradation. We systematically evaluated the effects of pH, temperature, substrate concentration, and reaction time on the activity of free and immobilized enzymes, along with their reusability and stability under operational conditions. The results demonstrate the potential of this functionalized SBA-15-based immobilization strategy as a highly efficient and sustainable platform for the bioremediation of tetracycline-contaminated environments. To the best of our knowledge, this is the first study to report the covalent immobilization of a metagenome-derived laccase (PersiLac1) onto imidazole-functionalized SBA-15 for the efficient degradation of tetracycline antibiotics, addressing the limitations of conventional laccase systems such as poor stability and high leaching. This innovative approach integrates the exceptional stability and catalytic potential of metagenomic enzymes with the precisely tailorable surface chemistry of SBA-15, resulting in a robust, reusable, and environmentally relevant biocatalyst.

## Materials and methods

### Reagents and materials

The recombinant laccase enzyme (PersiLac1) was identified and characterized in Agriculture Biotechnology Research Institute of Iran (ABRII)^19^. Chemicals including 2,2’-azino-bis (3-ethylbenzothiazoline-6-sulfonic acid ((ABTS), hydrogen peroxide (H_2_O_2_), copper (II) sulfate (CuSO_4_), Pluronic P123, tetraethyl orthosilicate (TEOS), sodium hydride (NaH; 95%), imidazole, (3-chloropropyl)triethoxysilane, and hydrochloric acid (37%), were purchased from Sigma Company. Solvents were also obtained from Sigma Company and used without any further purification. The antibiotics, tetracycline and doxycycline, were also obtained from Sigma Company.

### Preparation of surface functionalized SBA-15 (Im@SBA-15)

Based on our previous study^28^, from the reaction of freshly dried imidazole (2 g) and NaH 95% (0.77 g) in dry THF (60 mL) under an inert atmosphere, a suspension of sodium imidazolide was obtained. (3-chloropropyl)triethoxysilane (5.4 mL) was added to the prepared supension and the resulting mixture was refluxed for 30 h. After the removal of solvent under a reduced pressure, dry CH_2_Cl_2_ was added to remove the produced NaCl from the reaction mixture. CH_2_Cl_2_ phase was transferred to another flask and the volatiles were removed. In this step, N-(triethoxysilylpropyl)imidazole is obtained accompanied by unreacted starting materials. Dry toluene was added to the mixture, followed by transferring toluene phase to another flask and further solvent removing. Finally, the obtained product was washed with dry toluene to give pure n-(triethoxysilylpropyl)imidazole.

SBA-15 was silanized with n-(triethoxysilylpropyl)imidazole via previously reported procedures^33,34^. Freshly prepared SBA-15^35^ (1 g) and freshly synthesized n-(triethoxysilylpropyl)imidazole (1 mmol, 0.23 mL) were reacted in dry toluene under inert atmosphere. After 18 h reflux, the solid was filtered and washed with toluene and ethanol to remove unreacted n-(triethoxysilylpropyl)imidazole. The final product was dried to achieve surface functionalized SBA-15 material with imidazole tag, Im@SBA-15.

### Immobilization procedure, immobilization efficiency and leaching studies of LAC@Im@SBA-15

The laccase enzyme was dissolved in phosphate buffer, pH 6.0 and various amounts of support (Im@SBA-15) were added to the mixture followed by continuous agitation at room temperature for 60 min. The Bradford assay was used to determine protein concentration in the supernatant before (C_i_) and after (C_s_) immobilization and the immobilization efficiency and activity were calculated based on following equations^36^:

1 $${Immobilization~efficiency~}\left({\% } \right) = \frac{{C_{i} - C_{s} }}{{C_{i} }}{~} \times 100$$

2 $${Relative~activity~}\left({\% } \right) = \left( {\frac{A}{{A_{0} }}} \right) \times 100$$

Where A is the activity of enzyme after immobilization and A_0_ is the original activity before experiment^36^.

The leaching studies was performed by incubating immobilized laccase within a temperature bracket of 30 to 80 °C for a duration of 60 min, alongside a time-based incubation varying from 30 to 240 min at 25°C^37^. The enzyme loss due to leaching was calculated based on the following equation^37^:

3 $${Leaching~}\left({\% } \right) = \frac{{{Amount~of~protein~in~supernatant}}}{{{Amount~of~protein~immobilized}}} \times 100$$

The protein concentration was determined by using a BCA assay with bovine serum albumin (BSA) as standard. Immobilization efficiency values represent the mean ± standard deviation of three independent replicates.

### Laccase activity assay

For laccase activity determination, ABTS (2 mM) was used as substrate according to a previous study^38^. The reaction mixtures were incubated at 50 °C in phosphate buffer (50 mM, pH 6.0). The absorbance of solution was recorded at 420 nm using spectrophotometer (Ɛ_420nm_ = 36,000 M^− 1^ cm^− 1^). One unit of the laccase activity was expressed as the amount of enzyme oxidizing 1 µM of ABTS per minute under the standard assay condition.

In addition, the catalytic performance of free and immobilized laccase toward the target antibiotics tetracycline (TC) and doxycycline (DC) was evaluated by direct spectrophotometric monitoring of their degradation, as described in Sect.  3.6. These assays served as complementary activity tests to demonstrate substrate-specific performance beyond ABTS.

### Characterization of LAC@Im@SBA-15

UV-Vis spectroscopic measurements were conducted using UV-3100 SHIMADZU, UV-Vis-NIR recording spectrophotometer. The FTIR spectra were recorded in the medium infrared region from 4000 to 400 cm^− 1^ using a Shimadzu-IR. TGA was carried out for three samples using Netzsch-TGA 209 F1 under oxygen atmosphere, with a heating rate of 10 °C/min in the range of 20 to 800 °C. textural properties of particles was investigated via TEM images which were obtained using FILIPS CM300 electron microscope operating at 200 kV accelerating voltage.

### Biochemical characterization of free and immobilized laccase

#### Influence of pH and temperature on activity of free and immobilized laccase

To determine the effect of temperature on the activity of free and immobilized laccase, an extensive temperature spectrum, ranging from 30 to 80 °C was utilized in the potassium phosphate buffer (pH 6.0, 50 mM). In a similar vein, the pH impact on the activity of the free and immobilized laccase was assessed within pH range 4.0 to 9.0 using 50 mM of sodium citrate, potassium phosphate, and bicarbonate buffer solutions. In each of these investigations, the enzyme activity at varied pH values and temperatures was measured while the maximum activity being designated as 100%.

#### Efficiency of free and immobilized laccase in antibiotic treatment

The ability of free and immobilized laccase to remove tetracycline (TC) and doxycycline (DC) was evaluated under varying conditions of antibiotic concentration, temperature, and reaction time^39^.

To assess the time effect, 350 mg/L of antibiotics were treated with either free or immobilized laccase (0.2 U/mL) at 50 °C and pH 6.0 for up to 24 h. Samples were collected after 1, 2, and 24 h and analyzed spectrophotometrically. In addition, the effect of antibiotic concentration was tested by treating TC and DC solutions ranging from 25 to 300 mg/L with 0.2 U/mL laccase at 50 °C and pH 6.0 for 30 min. Antibiotic removal efficiency was calculated from the initial (C0) and final (Ct) concentrations according to Eq. (4), using standard calibration curves at 360 nm:

4 $${Antibiotic~removal~}\left({\% } \right) = \frac{{C_{0} - C_{t} }}{{C_{0} }} \times 100$$

To distinguish enzymatic degradation from nonspecific adsorption, control experiments were performed with functionalized SBA-15 nanoparticles (without laccase). The negligible adsorption observed (< 5%) was subtracted from the removal values obtained with the immobilized enzyme, thereby ensuring that the reported efficiencies reflect true enzymatic activity. The spectrophotometric monitoring at 360 nm was based on calibration curves prepared from pure TC and DC standards. Control results confirmed that absorbance changes were not due to nonspecific adsorption. This approach, while indirect, is widely applied in laccase degradation studies^11,39^and provides reliable quantification of antibiotic removal.

### Reusability of LAC@Im@SBA-15 in antibiotic removal

For measuring the reusability potential of immobilized laccase in elimination of antibiotics, reactions were conducted at 50 °C and pH 6.0 using 25 mg/L TC and DC through 10 cycles. After each reusability cycle, supernatant was separated via centrifugation and the laccase activity was quantified utilizing a spectrophotometer. The activity of the immobilized enzyme during the initial cycle was set as 100%.

## Results and discussion

### Immobilization of laccase in surface-functionalized SBA-15

**Fig. 1:**
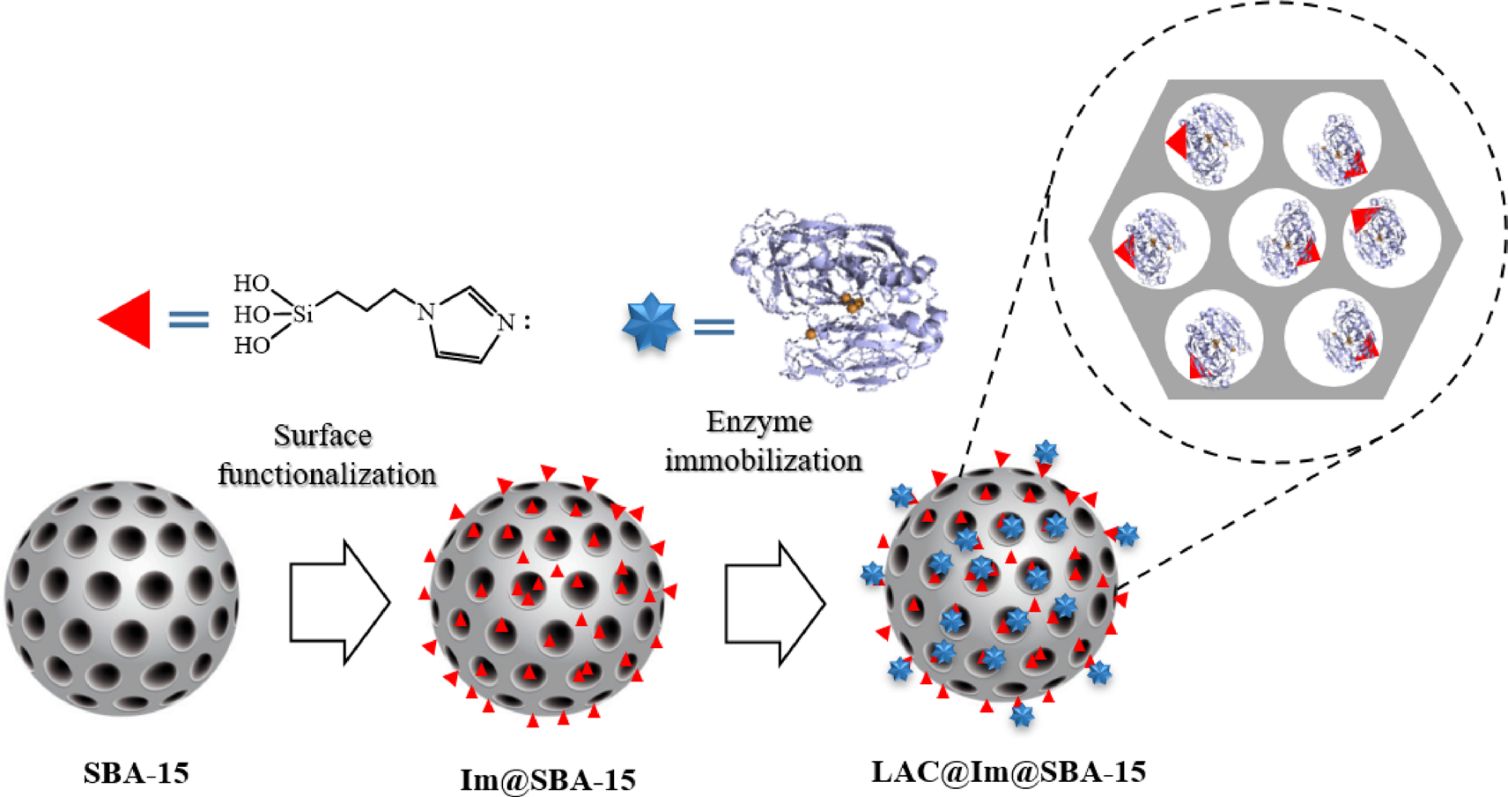
Synthetic procedure for the preparation of LAC@Im@SBA-15 nanosystem.

SBA-15 was obtained from the reaction of pluronic P123 and TEOS under acidic conditions following the reported procedure^35^. The resulting SBA-15 was chemically modified to have imidazole functional groups through post synthetic route^33,34^. From the reaction of SBA-15 with freshly prepared n-(triethoxysilylpropyl) imidazole, surface-functionalized material (Im@SBA-15) was obtained. In the next step, from the reaction of laccase with Im@SBA-15, a new nanosystem was afforded named as LAC@Im@SBA-15 (Fig. 1).

### Immobilization efficiency of LAC@Im@SBA-15

**Fig. 2:**
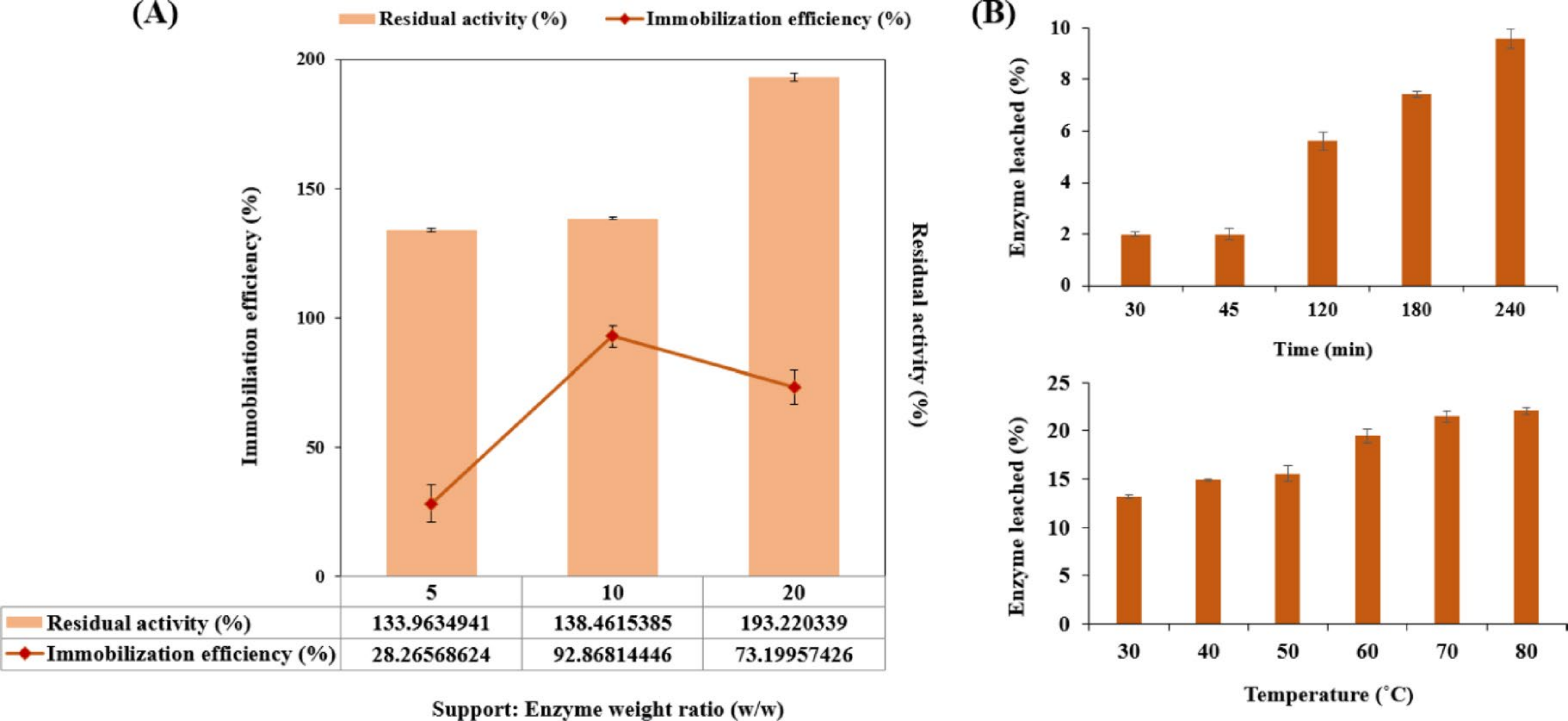
(**A**) Immobilization efficiency and residual activity at different amounts of support-to-enzyme ratios, (**B**) Enzyme leached out (%) at various incubation temperatures and durations. All values represent mean ± standard deviation (*n* = 3).

The immobilization efficiency of PersiLac1 was measured using various quantities of the support material Im@SBA-15 (1, 2, and 4 mg) in a fixed volume of enzyme solution and results are presented in Fig. 2A. The empirical evidence obtained revealed a direct correlation between the relative activity and the immobilization efficiency and the weight ratio of the support-to-enzyme (5 and 10). However, as the support-to-enzyme ratio exceeded 10 (w/w), the immobilization efficiency decreased that can be due to the restricted interactions between the nanocarrier and the enzyme molecules. Consequently, a support-to-enzyme ratio of 10 was identified as the optimal ratio to facilitate the immobilization of PersiLac1 in Im@SBA-15 material. This ratio was subsequently used for all assays performed within the scope of this study.

### Leaching studies of LAC@Im@SBA-15

Leaching studies are crucial for assessing the stability and operational efficacy of immobilized laccase systems. By conducting leaching studies, we ensured the maintenance of the desired enzymatic activity at various temperatures and times. As shown in Fig. 2B, 2% leached enzyme was obtained after 30 min of incubation at room temperature. This value was 9.57% after 240 min. Measuring the amount of leached enzyme under various temperatures, the leaching percentage was 12.18% was observed at 30 °C and then gradually increased to 22.06% at 80 °C. These results verified the potential of Im@SBA-15 nanocarrier as a favourable medium for enzyme immobilization, demonstrating that the robust interactions between the enzyme and support could mitigate enzyme loss, enhancing its stability against elevated temperatures and during storage. The same decrease in the leached enzyme was observed after immobilization of laccase into palladium nanoparticles^40^and MOF nanoparticles^41^.

### Characterization of LAC@Im@SBA-15

To analyse the physiochemical characteristics of new designed nanosystem (LAC@Im@SBA-15), Fourier transform infrared spectroscopy (FTIR), thermal gravimetric analysis (TGA), and transmission electron microscopy (TEM) techniques were applied.

**Fig. 3:**
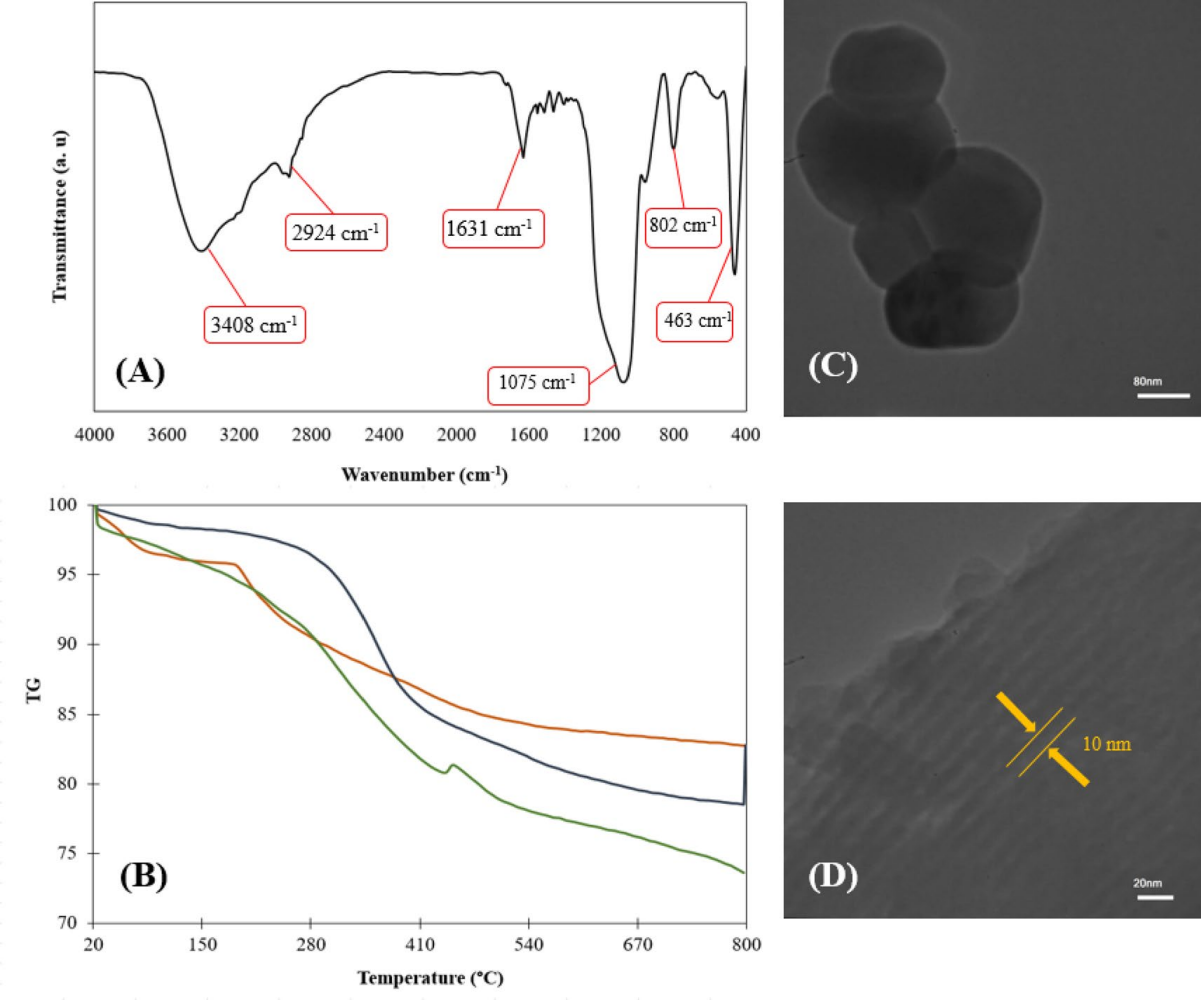
Physical characterizations. (**A**) FTIR spectra of LAC@Im@SBA-15, (**B**) TGA curves of SBA-15 (red), Im@SBA-15 (blue) and LAC@Im@SBA-15 (green), (**C** and **D**) TEM images of LAC@Im@SBA-15.

To investigate the successful preparation of LAC@Im@SBA-15, the FTIR analysis was employed (Fig. 3A). The FTIR spectrum of LAC@Im@SBA-15 confirms the successful synthesis, showing all characteristic bands of the core material consistent with previously reported spectra for SBA-15^34,42^. In particular, the C = N and C-Si stretching bands align with literature values, confirming successful grafting of imidazole moieties.^28^In the FTIR spectrum of LAC@Im@SBA-15, a broad band at 1075 cm^− 1^, accompanied by sharp band at 985 cm^− 1^ are distinctive vibrations attributed to Si-O-Si bonds. A broad signal at 3408 cm^− 1^ stands for the O-H bonds within the siloxane framework. The aliphatic and aromatic C-H stretching vibrations can be observed at 2924 and 2032 cm^− 1^, respectively. The characteristic bands located at 1631, 1552 and 802 cm^− 1^ are assigned for C = N, C = C and C-Si stretching vibrations, respectively. Consequently, the FTIR spectrum confirms the successful synthesis of LAC@Im@SBA-15.

To investigate the thermal behaviour of the materials, thermal gravimetric analysis (TGA) was studied (Fig. 3B). The functionalized SBA-15 (Im@SBA-15) showed about 2% weight loss that is due to the removal of water from its structure (Fig. 3B). TGA curve of Im@SBA-15 shows 15.37% weight loss that is related to the removal of surfactant template remained from extraction process. A major mass loss ∼500 to 800 °C are determined for removal separation of organic linkers. This data confirmed the successful surface functionalization of SBA-15^43^. TGA curve of LAC@Im@SBA-15 (Fig. 3C), exhibits a weight loss of 4.70% in the temperature below ∼120 °C corresponding to removal of adsorbed water. A weight loss (14.49%) between 120 and 400 °C is attributed to elimination of surfactant template remained from extraction process. The mass (7.75%) losses ranging from ∼400 to 800 °C are determined for removal of organic moieties and decomposition of laccase. Compared to the TGA curves of modified SBA-15 (Im@SBA-15)^44^, this data confirmed the successful enzyme immobilization.

To investigate the textural properties of new designed material, TEM images for LAC@Im@SBA-15 were recorded. As clearly observed in Fig. 3C, the spherical morphology has been detected for LAC@Im@SBA-15 material. In addition, TEM image presented in Fig. 3D apparently demonstrate the ordered parallel channels of new material indicating that hexagonal array of the mesostructure of the original SBA-15 silica is preserved throughout the chemical modification of the surface and consequently, enzyme immobilization.

### Biochemical properties of free and immobilized enzyme

**Fig. 4:**
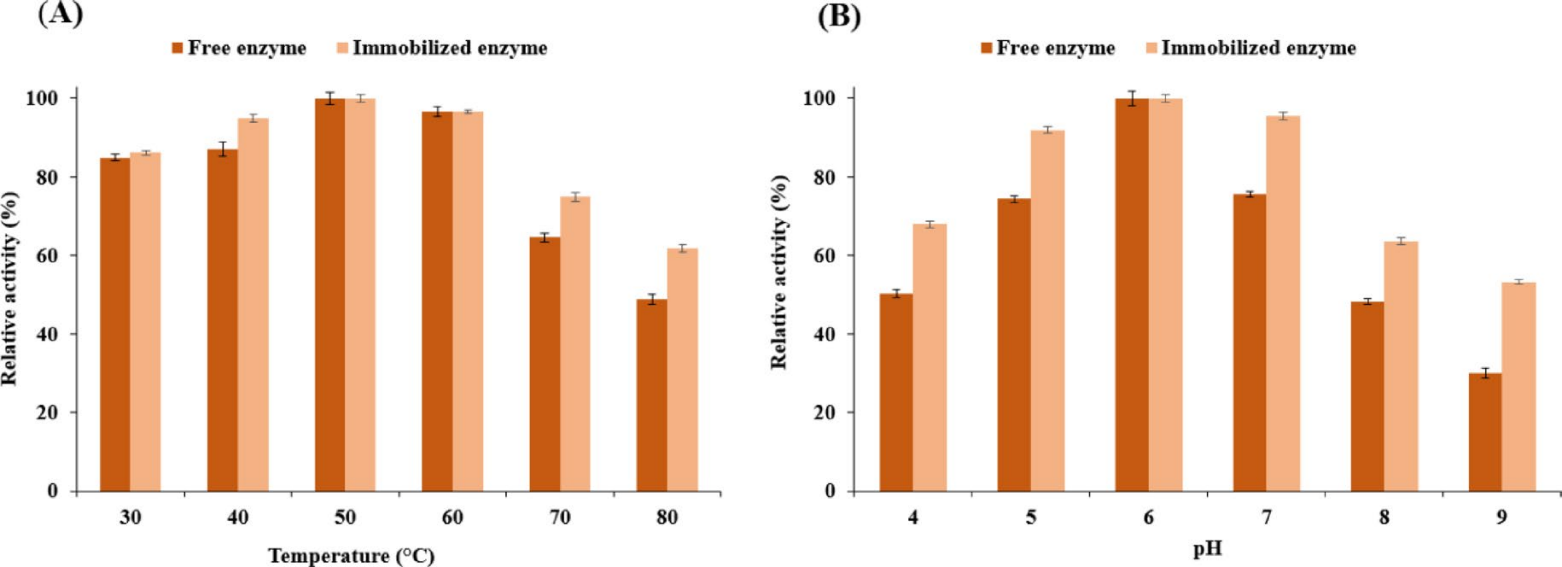
The influence of temperature and pH values on the activity of the free and immobilized laccase. All data are expressed as mean ± standard deviation (*n* = 3).

Selected factors influencing the activity of free and immobilized laccase, including the pH and temperature, were examined. For laccase in both forms (free and immobilized), optimal activity was obtained at 50 °C (Fig. 4A). At higher temperatures (70 °C and 80 °C), the activity of the free enzyme was between 48% and 64%. In contrast, the immobilized laccase exhibited higher activity when the temperature of the reaction system was higher than 70 °C showing 61 to 74% activity. Elevated temperatures have the potential to unravel protein molecules, leading to their aggregation which in turn deactivates enzymes. Possessing a high level of enzymatic activity under such extreme temperatures is a crucial trait for enzymes intended for a broad spectrum of applications. The findings of this study highlight the effectiveness of immobilization in improving the stability of enzymatic activity at high temperatures. These findings were better than the activity of immobilized laccase on the bimodal micro-mesoporous Zr-metal which showed nearly 70% activity when incubated at 70°C^45^. Moreover, these results indicated higher activity compared to magnetically separable laccase-biochar composite and an immobilized laccase on magnetic Fe_3_O_4_ particles coated with chitosan which exhibited less than 40% activity when incubated at 80°C^46^.

The activities of free laccase and immobilized one at pH range from 4.0 to 9.0 were also investigated (Fig. 4B). The immobilization increased laccase activity over this pH range compared with that of the native enzyme. Additionally, both free and immobilized laccase indicated maximum activity at pH 6.0. The immobilized enzyme retained more than 67% of its activity under acidic conditions (pH 4.0 to 5.0) and demonstrated 53–95% activity when incubated under neutral and alkaline conditions. In contrast, the free laccase showed much lower values under extreme alkaline and acidic conditions.

### Capability of free and immobilized laccase in elimination of antibiotics

Laccase-mediated degradation of antibiotics is a promising method in removal of certain antibiotics from contaminated environments through catalysing oxidative reactions and modifying or even fully degrading antibiotic molecules, thereby aiding in reducing their persistence and adverse impacts on the environment. Herein, we determined the influence of the free and immobilized laccase in the elimination of tetracycline (TC) and doxycycline (DC) varying the reaction condition including the temperature, pH and time, along with the concentration of the antibiotic.

The mechanism behind the laccase-mediated degradation of antibiotics like DC and TC involves enzymatic oxidative processes. However, the efficiency and the specific reactions can vary due to the different chemical structures of these antibiotics. It was stated that laccase can remove TC by oxidative decomposition and radical copolymerization. During these reactions, the hydroxyl group presented on the aromatic ring and the N-methyl functional group of TC molecule are major reactive parts^47^. Through the first pathway, the methyl group (C_6_) of TC is removed followed by hydroxylation reactions on C_5_ and C_11_. In the second route, epimerization happened at position C_4_ and form a product that could be decomposed by demethylation, deamination, dehydrogenation, and hydroxylation reactions. Similarly, three pathways have been suggested for DC degradation^11,48^.

In the first pathway, the oxidation of the double bonds at C_2_-C_3_ and C_11_-C_12_ occur, while in the second pathway, epimerization happen at position C_4_ and in third one, demethylation at C_4_ and C_6_ happens^48^.

Owing to the complex molecular structure of tetracycline family, laccases generally require an extended duration to effectively degrade them. In this research, we sought to evaluate the efficiency of both free and immobilized laccases in the degradation of TC and DC over a period of 24 h. All degradation experiments were performed with 0.2 U/mL of either free or immobilized laccase to allow direct comparison of kinetic data under equivalent enzymatic loading conditions.

**Fig. 5:**
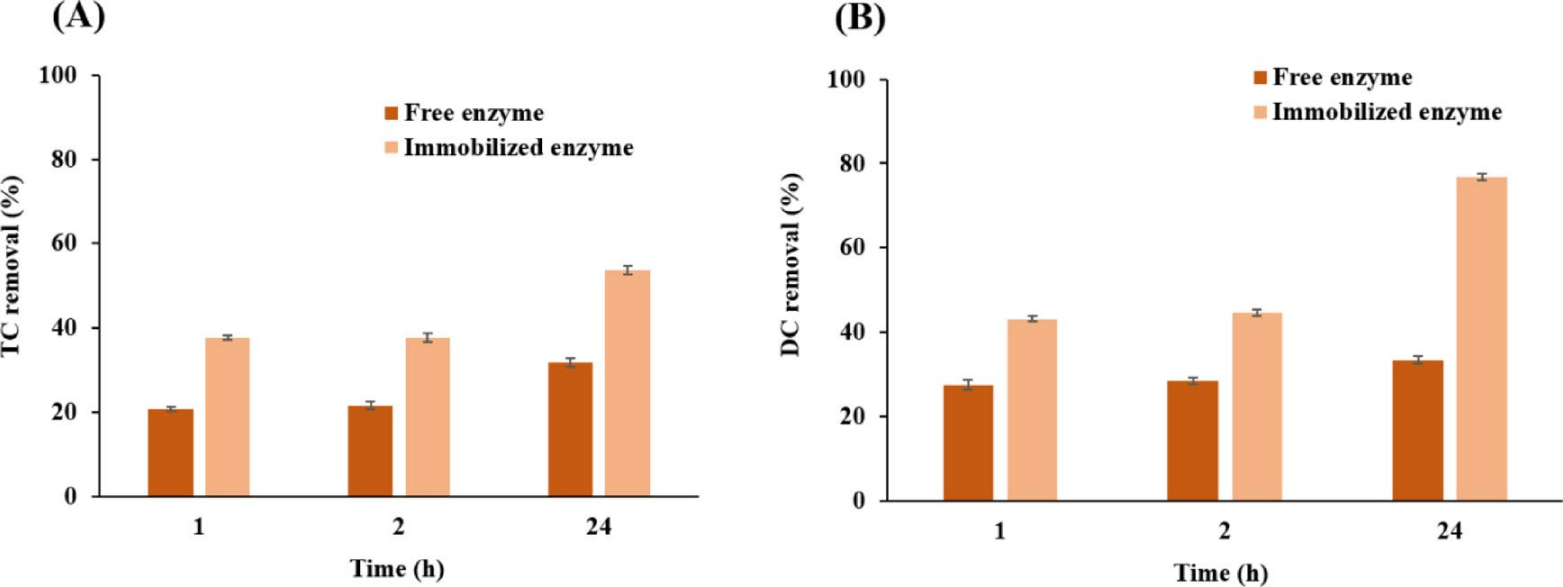
Time-dependent removal efficiency of tetracycline (TC) (**A**) and doxycycline (DC) (**B**) by free and immobilized laccase over a 24-hour period. Reactions were performed at pH 6.0 and 50 °C using 0.2 U/mL enzyme. Control experiments using functionalized nanoparticle (without enzyme) confirmed negligible antibiotic adsorption, which was subtracted from the total removal percentages to reflect true enzymatic degradation. All degradation experiments were performed with 0.2 U/mL of either free or immobilized laccase to allow direct comparison of kinetic data under equivalent enzymatic loading conditions and all data are expressed as mean ± standard deviation (*n* = 3).

As depicted in Fig. 5A, the outcomes of our assessment exhibited marked differences between the two states of the enzyme. At the outset hour of our exploration, the free laccase removed a 35.20% of TC. However, when the reaction time was extended to 24 h, the degradation efficiency increased, reaching a peak TC degradation of 36.07%. Beyond this 24-hour period, the degradation level remained largely stable, indicating that further extending the treatment time with laccase did not enhance the degradation. According to the results, immobilized laccase could degrade 47.40% of TC within 1 h. This degradation rate showed minimal change after 2 h. Nevertheless, an enhancement in degradation efficiency was recorded when TC incubated with immobilized laccase for 24 h achieving a TC removal rate of 53.65% (Fig. 5A).

In the case of DC, free laccase demonstrated a removal of 18.06% after 1 h which reached to its maximum of 33.39% after 5 h. Additionally, immobilized laccase was more effective in eliminating DC. A DC removal efficiency of 43.11% after a 1 h, and this efficiency increased to 76.69% following a 24-hour treatment period.

These data revealed that the immobilized laccases demonstrated a markedly higher capability to degrade TC and DC in a more efficient manner. Moreover, with free laccase, the elimination of TC is more pronounced within the initial two hours. However, after 24 h, the free enzyme demonstrated nearly equal efficiency in degrading both DC and TC. In contrast, the immobilized enzyme initially showed a higher capacity to remove TC during the early stages of hydrolysis. However, over an extended period of 24 h, the immobilized enzyme proved to be significantly more effective in eliminating DC, surpassing its efficiency in TC removal.^49^This suggests that while both forms of the enzyme (free and immobilized), are effective, their long-term degradation capabilities vary depending on the type of compound and duration of treatment.

**Fig. 6:**
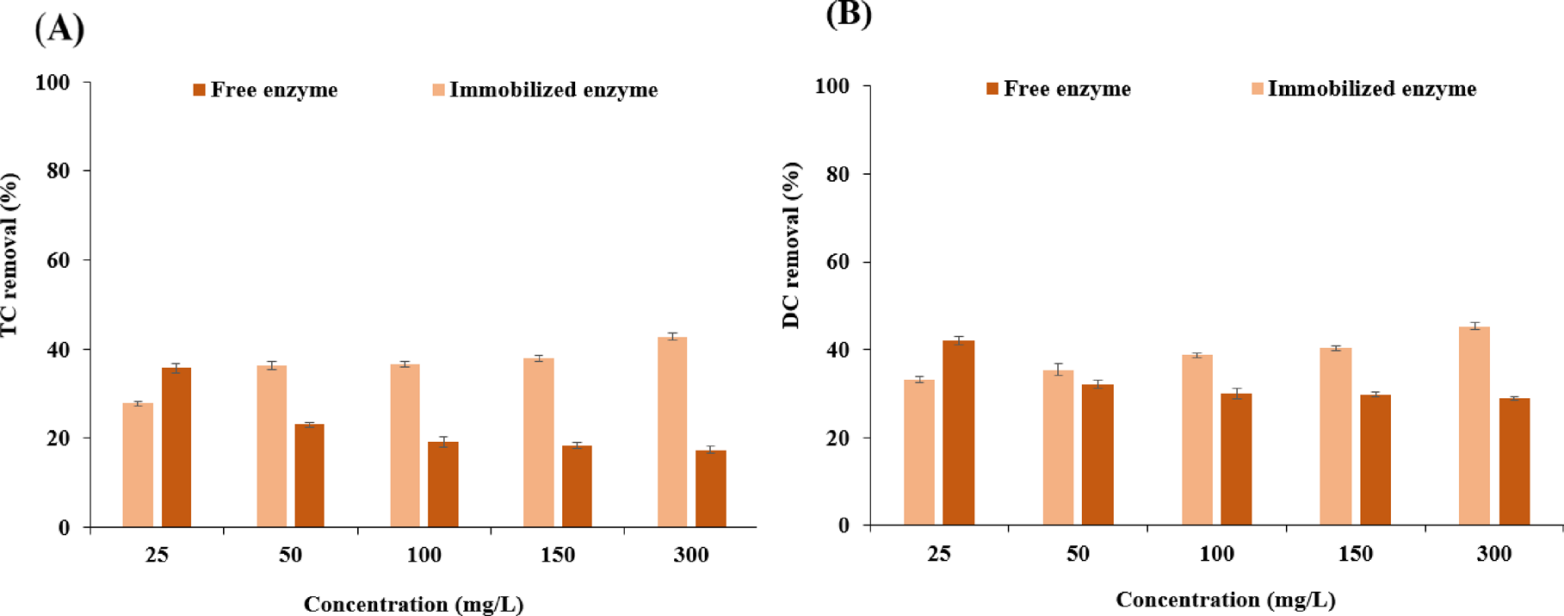
Effect of antibiotic concentration on the removal of tetracycline (TC) (**A**) and doxycycline (DC) (**B**) in the range of 25–300 mg/L by free and immobilized laccase. Reactions were conducted at optimal conditions (pH 6.0, 50 °C, 0.2 U/mL laccase, 30 min). Removal efficiencies were corrected by subtracting background adsorption determined in control tests using enzyme-free nanoparticles. All data are expressed as mean ± standard deviation (*n* = 3).

The efficacy of the removal of TC and DC was examined across a concentration spectrum of 25 to 300 mg/L under optimal operational conditions as shown in Fig. 6A. At the minimal concentration of 25 mg/L, free laccase displayed a removal efficiency of 35.69% for TC and this value decreased over time to 17.38% at the highest concentration (300 mg/L). In comparison, the immobilized variant demonstrated enhanced effectiveness at higher TC concentrations. As shown in Fig. 6A, the free enzyme was more efficient at removing TC at lower concentrations. However, the immobilized laccase showed greater efficiency, while increasing the concentration of TC, achieving a 42.80% removal rate in the presence of 300 mg/L TC. In contrast, free laccase exhibited a noticeable decline in activity at higher TC concentrations, leading to reduced removal efficiency. This contrast highlights the robustness of immobilized laccase, which maintained a high efficiency even in the presence of elevated concentrations of antibiotics. The data suggest that while both forms of laccase (free and immobilized), are effective, the immobilized laccase is more capable in environments with higher contaminant levels.

Furthermore, we evaluated the impact of free and immobilized enzymes on the removal of various DC concentration. Figure 6B reveals that the degradation efficiency for the free enzyme was 42.09% and gradually decreased to 28.96%. On the contrary, when the immobilized laccase was applied, the DC removal percentage was found to be 18.28% at the lowest concentration of DC while this value enhanced up to 43.96% under 300 mg/L of DC. This set of outcomes underscores the advantages of the immobilized laccase, especially when tasked with remediation in environments with high antibiotics concentrations.

These findings further highlight that the immobilized laccase was more adept at hydrolysing both TC and DC, particularly at higher concentrations of these compounds. While free laccase was effective in antibiotic removal, immobilized laccase excelled with the increased antibiotic concentration. This enhanced performance of immobilized laccase can be attributed to its superior stability, which allows it to resist the inhibitory effects that often accompany increasing antibiotic concentrations. This resilience not only underscores its efficiency in degrading these compounds but also points to its potential utility in applications involving antibiotic treatment, especially in scenarios where high contaminant concentrations are a challenge. This ability to maintain effectiveness in more demanding environments makes the present immobilized laccase nanosystem a promising tool in the field of environmental remediation and antibiotic degradation.

TC and DC removal ability of the free and immobilized laccase is compared with reported laccases in literature (Table 1). Upon comparison with the data presented in Table 1, it was observed that free enzyme demonstrated the ability to degrade 44 to 52% of 50 mg/L TC and DC within 2 h. While most laccases in literature need more time to remove lower concentration of these antibiotics. In the referenced literature, the immobilized laccase enzymes exhibited a degradation efficiency up to 98% for over varying durations of 3 to 48 h. However, in our experimentation, the designed immobilized laccase nanosystem removed 200 mg/L of TC within just 2 h with an efficiency percentage of 42–46%. A distinguishing aspect of our research is the lower concentration of laccase utilized. While most referenced studies employed laccase concentrations of 0.5 U/mL or higher, our experiments were conducted with a notably lower concentration of 0.2 U/mL for both free and immobilized laccase. Despite the lower enzyme concentration, notable DC and TC removal was achieved within a shorter timeframe, underlining the effectiveness of laccase in the degradation process.

To ensure that the observed removal of TC and DC was due to enzymatic degradation rather than nonspecific adsorption, we conducted control experiments using functionalized SBA-15 nanoparticles without any enzyme. The results demonstrated that only a negligible amount of antibiotic was adsorbed by the carrier alone (less than 5%)^50^. This minor adsorption contribution was subtracted from the removal percentages obtained with the immobilized enzyme system. Therefore, the reported efficiencies of TC and DC elimination reflect the true catalytic performance of the immobilized laccase. This distinction is crucial for confirming the biodegradation capability of the immobilized system, especially in comparison with previously reported systems that often neglect to account for the adsorption background.

**Table 1:** Comparative data on the enzymatic degradation of Tetracycline (TC) and Doxycycline (DC) by various free and immobilized laccase systems.

Laccase source/Microorganism	Laccase concentration (U/mL)	Enzyme form	Antibiotic concentration (mg/L)	Antibiotic	pH/Temperature (°C)	Time (h)	Antibiotic removal (%)	Mediator	Real matrix	References
*Bacillus amyloliquefaciens*	0.5	Free	100	TC	pH 7.0/ 30	12	37.5	None	Buffer	^47^
*T. versicolor*	-	Immobilized	20	TC	pH 6.0/ 25	24	56	HBT	Buffer	^51^
*T. versicolor*	0.5	Free	100	TC	pH 7.0/ 20	18	78	HBT	Buffer	^51^
*T. versicolor*	0.5	Immobilized	10	TC	pH 6.0/ 25	24	85	HBT	Buffer	^52^
*Cerrena sp*	40	Free	100	TC	pH 7.0/ 25	12	80	None	Buffer	^53^
*Cerrena sp*	20	Immobilized	100	TC	pH 7.0/ 25	48	65	None	Buffer	^53^
*T. versicolor*	-	Immobilized	20	TC	pH 4.5/ 30	3	98	None	Buffer	^54^
*T.versicolor*	-	Free	20	TC	pH 6.0/ 25	24	56	None	Buffer	^51^
*Riemerella sp.* and *Acinetobacter sp*	-	-	50	DC	37	48	95	None	Real (WW)	^10^
*T. versicolor*	10	Free	40	DC	pH 4.5/ 30	4	> 80	None	Buffer	^6^
*Trametespolyzona*	1	Free	25	DC	pH 4.5/ 50	24	100	None	Buffer	^55^
*Trametes versicolor*	-	Free	10	DC	pH 4.0/ 25	2	100	None	Buffer	^1^
*Metagenome-derived*	0.2	Free	50	TC	pH 6.0/ 50	2	52.80	None	Buffer	This study
*Metagenome-derived*	0.2	Immobilized	200	TC	pH 6.0/ 50	2	42.02	None	Buffer	This study
*Metagenome-derived*	0.2	Free	50	DC	pH 6.0/ 50	2	44.88	None	Buffer	This study
*Metagenome-derived*	0.2	Immobilized	200	DC	pH 6.0/ 50	2	46.1	None	Buffer	This study

The table summarizes key experimental parameters, including enzyme source, dose, reaction time, temperature, and matrix type (buffer vs. real wastewater). Additionally, the presence of redox mediators is reported where applicable. These details enable a more realistic and contextualized comparison across studies, highlighting practical performance versus lab-scale optimizations.

While Table 1 enables contextual comparison, reported removal efficiencies vary widely because of differences in operating conditions and system design. In particular, initial antibiotic concentration and reaction time jointly shape apparent removal: higher loadings can impose diffusion constraints and/or inhibitory effects, whereas longer contact times may increase endpoint removal values. The use of redox mediators (e.g., HBT) is another major source of variability, as mediator-assisted laccase systems often accelerate oxidation relative to mediator-free setups. Moreover, enzyme format (free vs. immobilized) and support properties can affect active-site accessibility, mass transfer, enzyme leakage, and operational stability. Matrix complexity (buffer vs. real wastewater) may further modify outcomes through competitive adsorption, radical quenching, and changes in substrate bioavailability. Accordingly, cross-study comparisons based solely on “% removal” should be interpreted cautiously unless these variables are aligned or explicitly considered.

In this context, it should be noted that the tetracycline concentrations examined here (up to 200 mg/L) mainly reflect high-strength contamination scenarios (e.g., pharmaceutical/hospital effluents) rather than typical environmental waters. These elevated levels were selected to assess the maximum-capacity performance and robustness of immobilized PersiLac1 under stringent loading. However, because the experiments were conducted in model solutions, constituents in real wastewaters (e.g., natural organic matter, salts, suspended solids, and co-contaminants) may influence activity and removal efficiency. Therefore, extrapolation to real environmental conditions should be made with caution, and future work should validate performance in real wastewater matrices and at environmentally relevant tetracycline concentrations to quantify matrix effects and confirm field applicability.

In addition, although enzymatic oxidation reduced the parent tetracycline/doxycycline concentrations, removal of the parent compound does not inherently demonstrate complete detoxification. Oxidative transformation can produce intermediate/byproduct species that may retain biological (including antibacterial) activity or present residual ecotoxicity. Therefore, while the present study demonstrates promising laboratory-scale performance, confirmation of safety and completeness of degradation—especially prior to real-wastewater deployment and industrial-scale implementation—requires toxicity-oriented validation in addition to chemical analytics.

### Reusability of LAC@Im@SBA-15 in antibiotic removal

**Fig. 7:**
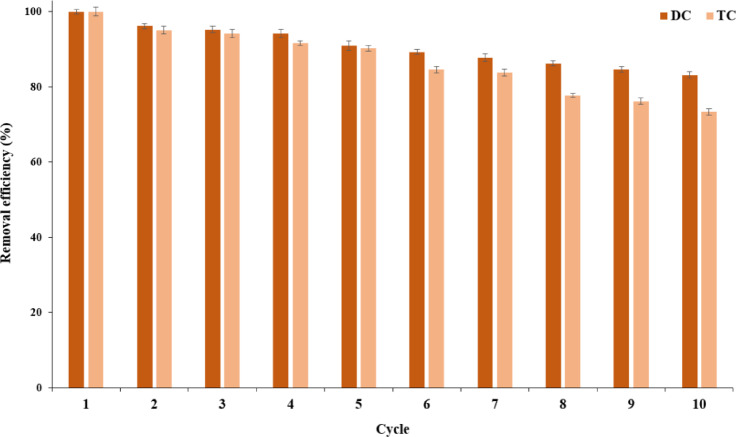
Reusability of immobilized PersiLac1 in removal of 25 mg/L TC and DC over 10 cycles. All data are expressed as mean ± standard deviation (*n* = 3).

The significance of the reusability of immobilized laccase in antibiotic removal from wastewater extends beyond its environmental impact, serving as a key factor in sustainable and cost-effective water treatment strategies^56^. This study delved into the efficacy of immobilized PersiLac1 in eliminating TC and DC over ten cycles, with detailed results depicted in Fig. 7. The immobilized enzyme demonstrated high antibiotic removal efficiency, nearing 100% in the first few cycles. However, a notable limitation was the progressive decline in enzymatic activity observed in subsequent reuse cycles, which may be attributed to partial enzyme inactivation, structural instability of the support, or enzyme leaching.

In addition, a gradual decrease in efficiency was observed with subsequent cycles, with efficiencies of 83.18% for DC and 73.35% for TC. This pattern highlights not only the initial robustness of the immobilized laccase in targeting these specific antibiotics but also points to the decline in activity over extended use. By demonstrating the potential of immobilized laccase in this domain, the study contributes to the development of more eco-friendly and efficient water treatment methods. Nevertheless, the observed decline in performance over repeated cycles highlights a critical limitation for real-world applications, and future efforts should focus on enhancing the operational stability of immobilized systems through improved immobilization techniques or protective coatings.

Same high removal efficiency of laccase in TC was observed previously by immobilizing of *Bacillus subtilis* laccase into the copper-Trimesic acid framework^57^. In another work a bacterial laccase from *Bacillus amyloliquefaciens* was immobilized on hybrid nanoflowers and showed less than 80% removal efficiency of tetracycline antibiotics after 5 cycles^47^. Moreover, immobilization of laccase on hollow mesoporous carbon nanospheres possess efficient removal of tetracycline over multiple cycles^54^.

Although the immobilized PersiLac1 retained > 70% of its initial activity after 10 consecutive cycles, a gradual decline was observed. This loss may arise from multiple, non-mutually exclusive factors: (i) progressive enzyme inactivation under repeated operational stresses (e.g., thermal exposure and oxidative stress during laccase-catalyzed reactions), (ii) partial surface/pore fouling of SBA-15 by adsorbed tetracycline and oxidation byproducts, including possible polymeric deposits that can reduce effective mass transfer and active-site accessibility, (iii) mechanical loss of the immobilized biocatalyst during separation/washing steps between cycles, and (iv) minor enzyme leaching or conformational changes over repeated use, although covalent anchoring is expected to minimize leaching. To further enhance stability, future studies aimed at improving overall efficiency may consider optimizing the immobilization chemistry to promote multipoint attachment and a more favorable enzyme orientation (e.g., by using spacer arms), applying mild washing/regeneration protocols to remove adsorbed byproducts, and employing thin protective coatings that reduce fouling without compromising diffusion.

## Conclusion

Tetracycline antibiotics represent a serious environmental hazard, threatening biodiversity and ecosystem function by disrupting microbial communities in both soil and aquatic habitats. Effective removal of these persistent contaminants is therefore essential to safeguard ecological health. In this study, we developed a robust and efficient biocatalyst by covalently immobilizing a metagenome-derived laccase (PersiLac1) onto imidazole-functionalized SBA-15 mesoporous silica. This functionalization strategy significantly reduced enzyme leaching and preserved catalytic activity over extended operational periods.

The immobilized laccase exhibited high degradation efficiencies for both doxycycline (DC) and tetracycline (TC), maintained strong performance even at elevated antibiotic concentrations, and demonstrated resilience under diverse environmental conditions. Furthermore, it retained more than 70% of its initial activity after ten consecutive reuse cycles, underscoring its operational stability.

These findings suggest that the developed immobilized laccase system demonstrates promising performance under controlled laboratory conditions for the removal of tetracycline and doxycycline. Although no real wastewater matrices were tested in this study, the results provide a valuable foundation for future investigations. However, because the experiments were performed in model solutions, real-world applicability remains to be demonstrated, particularly in complex wastewater matrices where salts, natural organic matter, suspended solids, and co-contaminants may influence apparent activity and removal efficiency.

Further validation using real effluents and complex environmental samples will be essential to confirm the practical applicability of the system in wastewater treatment scenarios.

Future work should validate the performance of the immobilized PersiLac1 system in real wastewater matrices and at environmentally relevant tetracycline concentrations, and should further assess long-term operational stability and scalability prior to practical implementation.

## Data Availability

All data generated or analysed during this study are included in this published article.
